# Genetic diversity and fine-scale spatial genetic structure of European beech populations along an elevational gradient

**DOI:** 10.1038/s41437-025-00776-8

**Published:** 2025-06-26

**Authors:** Ourania Grigoriadou Zormpa, Selina Wilhelmi, Boban Vucetic, Mihnea-Ioan-Cezar Ciocîrlan, Markus Mueller, Elena Ciocîrlan, Alexandru Lucian Curtu, Mehdi Ben Targem, Henning Wildhagen, Oliver Gailing, Katharina B. Budde

**Affiliations:** 1 Faculty of Forest Sciences and Forest Ecology, Forest Genetics and Forest Tree Breeding, University of Göttingen, Göttingen, Germany; 2 Center for Integrated Breeding Research (CiBreed), University of Göttingen, Göttingen, Germany; 3 Faculty of Silviculture and Forest Engineering, Transilvania University of Brașov, Brașov, Romania; 4 National Institute for Research and Development in Forestry (INCDS) Marin Drăcea, Brașov, Romania; 5 HAWK University of Applied Sciences and Arts, Faculty of Resource Management, Göttingen, Germany; 6 Center of Biodiversity and Sustainable Land Use (CBL), University of Göttingen, Göttingen, Germany; 7 Northwest German Forest Research Institute, Hann Münden, Germany

**Keywords:** Natural variation in plants, Genetic variation

## Abstract

Differences in environmental conditions can shape the level and distribution of intraspecific genetic variation between and within populations. Elevational gradients are characterised by strong variation in environmental conditions on a short spatial scale and provide an ideal setting to study the spatial distribution of genetic diversity. Therefore, we investigated the genetic diversity, fine-scale spatial genetic structure (FSGS) and spring phenology (bud burst) as a proxy for flowering of five European beech (*Fagus sylvatica* L.) populations along an elevational gradient, ranging from about 550 m to 1450 m a.s.l. in the Romanian Carpathians. Using microsatellite and genome-wide single nucleotide polymorphism (SNP) markers, we observed a slight decrease in genetic diversity with increasing elevation and low population differentiation. Furthermore, levels of FSGS decreased with elevation along the gradient. We could not detect any significant effects of spring phenological traits on the level of FSGS probably because many different environmental factors and processes vary over the years and contribute to shaping the FSGS. The slightly lower genetic diversity in high elevation populations may indicate stronger drift effects and could be due to the marginal ecological conditions and the lower abundance of beech. However, in these stands with less competing crowns and a more open forest structure, pollen dispersal might be longer ranging in this wind pollinated species which could contribute to a weaker FSGS. The knowledge about the level and structure of genetic variation along environmental gradients is crucial to inform forest and conservation management especially in the face of climate change.

## Introduction

Genetic variation plays a fundamental role in ecological and evolutionary processes of natural populations (Hughes et al. 2008). High levels of genetic variation within populations provide greater adaptive potential making species less vulnerable to changing conditions (Alberto et al. 2013). The distribution of genetic variation can therefore inform conservation priorities and, in the case of forest tree species, forest management (Fady et al. 2020). The effects of different environmental conditions on the level and distribution of intraspecific genetic variation are of great interest. Core populations at the centre of a species’ distribution often harbour higher genetic diversity than geographically marginal populations at the edge of the species distribution (Eckert et al. 2008; Theraroz et al. 2024). This is typically explained by larger effective population sizes, which reduce genetic drift, and efficient gene flow between populations in the core area compared to smaller and more fragmented populations at the periphery (e.g., Pandey and Rajora 2012a). However, ecologically marginal populations, not necessarily located at the periphery of a species’ distribution, but growing under harsher environmental conditions, may also harbour lower genetic diversity (Soulé 1973; Pironon et al. 2017). These populations, in less suitable environments, display lower effective population size and experience stronger selection pressures and stronger drift effects than populations growing in optimal conditions (Kawecki 2008). Elevational gradients offer a unique setting to study the effect of different environmental conditions on the level and distribution of genetic diversity, as they are characterised by steep changes in environmental conditions over short spatial scale. For example, while tree populations of temperate deciduous tree species at low or mid elevations in Europe may experience close to optimal conditions, populations of the same species at higher elevations often face extreme conditions similar to those at their northern latitudinal range limits (Randin et al. 2013). Moreover, environmental conditions are currently changing due to global climate change, which will alter species distributions and population dynamics in future generations also along elevational gradients (Vitasse et al. 2021).

The spatial genetic structure, i.e., the non-random distribution of alleles and genotypes in space, is influenced by mutation, gene flow, selection and drift (Wright 1949). Between subpopulations of a species distinct selection pressures, gene flow and genetic drift can cause differences in allele frequencies which determine the level of population differentiation (Ehrlich and Raven 1969). Gene flow, mediated by both pollen and seed dispersal, is a major factor determining the genetic structure of plant populations. The level of gene flow is determined by the population history and life history traits such as the mating system and the mode of pollen and seed dispersal of a species (Petit and Hampe 2006). Previous studies have reported wide ranging pollen flow over distances of 5 to ~ 10 km for wind-pollinated tree species (e.g., Bacles et al. 2005; Robledo-Arnuncio and Gil 2005; Jiménez-Ramírez et al. 2021) whereas long distance seed dispersal events are rare (e.g., Bacles et al. 2006). Long distance gene flow typically promotes high levels of genetic diversity within populations and low levels of genetic differentiation between populations. However, environmental conditions, such as a rugged topography, can contribute to impeding gene flow and can thereby increase differentiation between tree populations even at the local spatial scale (Moracho et al. 2016). Differences in environmental conditions along elevational gradients are known to influence spring phenology, with bud burst and flowering starting earlier in low-elevation and later in high-elevation populations (Vitasse et al. 2009; Pellerin et al. 2012). Limited overlap in flowering phenology between populations along an elevational gradient can restrict gene flow by pollen dispersal and lead to population differentiation (Jordano and Godoy 2000).

In addition to genetic diversity and population differentiation, also the fine-scale spatial genetic structure (FSGS) within populations has been suggested as a parameter for genetic monitoring in trees (Major et al. 2021). The strength of FSGS has been studied in a variety of plant and tree species and describes the decrease in relatedness between individuals with increasing distance (Heywood 1991; Vekemans and Hardy 2004; Goncalves et al. 2022). It can affect future generations because a strong FSGS increases the probability of mating of related individuals (Finkeldey and Ziehe 2004), which promotes the risk of inbreeding depression due to increased homozygosity and loss of genetic diversity within populations (Porcher and Lande 2016). The strength of FSGS can vary between populations of the same species, and despite numerous studies (Torroba-Balmori et al. 2017; Mosca et al. 2018; Major et al. 2021), the factors driving these intraspecific differences remain poorly understood. In some tree species, marginal populations showed stronger FSGS than core populations (e.g., Gapare, Aitken (2005); Pandey and Rajora 2012b; Götz et al. 2022) which seems to be related to lower population density and isolation in peripheral and ecologically marginal populations. Abiotic conditions, such as temperature and precipitation, can influence spring phenology traits, such as bud burst and flowering, and alter gene flow patterns or recruitment success, thereby shaping the FSGS within populations (De-Lucas et al. 2009; Mosca et al. 2018; Hirao and Kudo 2008). In *Larix decidua* Mill., variability in spring temperature, likely reflecting the risk of late frost events, which can affect the flowering buds, pollination and seed maturation, was correlated with FSGS while this was not the case in other conifer species (Mosca et al. 2018). Other studies in *Larix decidua* and *Abies alba* Mill. showed that FSGS is affected by elevation. High elevation and recently recolonised populations had stronger FSGS compared to populations at low elevations (Nardin et al. 2015; Major et al. 2021). However, studies that investigate the effects of different environmental conditions or phenology on the FSGS in forest tree species are rare.

In the present study, we investigate the genetic diversity, population differentiation and FSGS in naturally regenerated populations of European beech (*Fagus sylvatica*) along an elevational gradient in the Romanian Carpathians, near the city of Brașov. Along the elevational gradient in our study area, the proportion of beech decreases with altitude as other tree species, such as *Abies alba* and *Picea abies* (L.) H. Karst, become more frequent. In analogy to marginal populations growing in suboptimal environmental conditions, we consider high-elevation populations growing near the elevation range limit of *F. sylvatica* as ecologically marginal. We hypothesize that these populations will harbour lower genetic diversity and stronger FSGS than low-elevation populations. Temporal differences in spring phenology in *F. sylvatica* populations along elevational gradients have been observed (Ciocîrlan et al. 2024a; Gauzere et al. 2013) and such differences can cause assortative mating and affect gene flow (Fox 2003). In European beech, bud burst is considered a good proxy for flowering phenology (Millerón et al. 2012; Gauzere et al. 2013) as pollen release typically overlaps with the last bud burst phase (Millerón et al. 2012). We therefore re-analysed the bud burst data previously characterised by Ciocîrlan et al. (2022, 2024a, b), with a particular focus on the duration of bud burst and the last bud burst stage. We hypothesised that different environmental conditions along the elevational gradient could influence the timing of bud burst and thus the synchrony of flowering, which in turn could affect gene flow between and within populations and shape the FSGS.

To investigate these hypotheses regarding genetic diversity and FSGS along the elevational gradients in *F. sylvatica*, we used a combination of molecular markers. Traditionally, SSR (simple sequence repeat) markers have been used to assess genetic diversity and FSGS, however these studies are usually limited to a low number of markers. High throughput sequencing is now enabling the genotyping of high numbers of genome-wide SSR (e.g., Lepais et al. 2020) and SNP markers (e.g., Davey et al. 2011). In order to test if a higher number of markers could reveal further insights, we used 12 nuclear SSR and several thousand neutral SNP markers to assess the population genetic structure and FSGS, as well as the genetic diversity in five European beech populations ( ~ 500 individual trees) along the elevational gradient.

## Materials and Methods

### Study species

European beech is one of the most common and wide-ranging tree species in Europe. It is wind-pollinated and its single seeded beechnuts (arranged in pairs within a cupule) are dispersed by gravity and animals. Pollen dispersal (28–55 m, Oddou-Muratorio et al. 2010; 2011; 62–162 m, Millerón et al. 2012; 80–184 m, Piotti et al. 2013;) is more efficient than fruit and seed dispersal (up to ~10 m, Oddou-Muratorio et al. 2011; 12–25 m Bontemps et al. 2013). Pollen flow between different populations is often high (e.g., 63–68%, Oddou-Muratorio et al. 2010; 75–81%, Piotti et al. 2012). Therefore, populations of *F. sylvatica* are typically characterised by high levels of genetic diversity and low population differentiation between populations (Postolache et al. 2021; Stefanini et al. 2022). At the range-wide scale the species harbours three main gene pools (Postolache et al. 2021) which reflect the postglacial recolonization history in Europe (Magri et al. 2006). Female flowers are receptive a few days before male flowers of the same tree (Nielsen and Schaffalitzky de Muckadell 1954) and high elevation populations start flowering later than low elevation populations (Gauzere et al. 2013; Ciocîrlan et al. 2022, 2024a, 2024b). This phenological lag contributed to high long-distance pollen flow between and within populations of beech along an elevational gradient at Mont Ventoux (Gauzere et al. 2013).

### Study system and field work

**Fig. 1:**
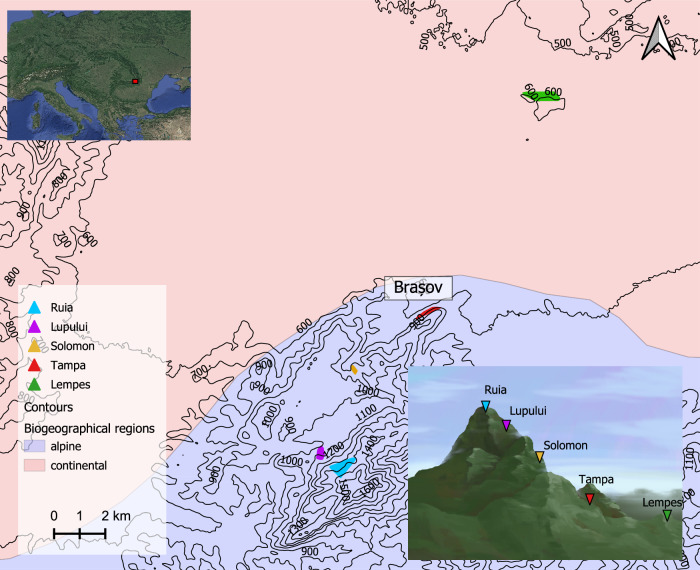
Geographic location of the five European beech populations sampled along an elevational gradient in the Romanian Carpathians. Carpathian montane forests cover the southern alpine and continental region from 500 to 1500 m (a.s.l.) (Olson et al. 2001; EEA 2021).

We selected five naturally regenerating European beech populations, along an elevational gradient in Romania, near the city of Brașov. These mixed forest stands grow in the south-eastern Carpathian Mountains, between 550 m and 1450 m above sea level (a.s.l.) (Table 1, Fig. 1). European beech is dominant in lower elevations (e.g., in Lempes at 550–650 m a.s.l.) with a proportion of about 70%. *Carpinus betulus* L., *Acer platanoides* L., *Fraxinus excelsior* L., among others, are some of the tree species that coexist with beech in lower elevations. At higher elevations, particularly in Ruia and Lupului, other species such as *Picea abies* and *Abies alba* become more common. The density of beech decreases with increasing elevation down to a proportion of 10–20% at the highest elevation (Ruia, 1300–1450 m a.s.l., Table 1). An exception of this trend is Solomon at intermediate elevation with a very high proportion of beech (70–80%). In Ruia, the highest elevation population, the forest shows a patchy structure with gaps. The slope was very steep in all sites, except for the lowest elevation population (Lempes). All populations form part of a continuous forest cover along the Postavarul mountain with the exception of the lowest elevation population (Lempes) which is more isolated.

**Table 1:** Characteristics of the five European beech study populations located along the elevational gradient in the Romanian Carpathians. E, East; N, North and SW, Southwest exposition on the top of the hill.

	Ruia	Lupului	Solomon	Tampa	Lempes
Position	45°34‘39“N	45°34‘58“N	45°36‘59“N	45°38‘21“N	45°43‘34“N
	25°33‘18“E	25°32‘38“E	25°33‘37“E	25°35‘46“E	25°39‘18“E
Elevation above sea level (m)	1300–1450	1000–1150	800–1000	650–750	550–650
Exposition	N	N	N-SW	N	N
Mean annual temperature (°C)	3.5	5.2	6.2	7.2	7.5
Mean annual precipitation (mm)	1023	957	855	791	712
Soil type	Rendzina	Rendzina	Rendzina	Rendzina	Luvisol
Proportion of European beech	~ 10–20%	~ 20–30%	~ 70–80%	~ 40–50%	~ 70%

We have reviewed data on the silvicultural history of the region and to the best of our knowledge, all populations have established naturally. Almost no silvicultural operations have been carried out in the sampled stands during the last decades, as two sites (Lempes and Tampa) are located in natural protected areas (Natura 2000) and the other three sites (Solomon, Lupului and Ruia) are located close to the city of Brașov or to a ski resort (Ruia). In 1946, a fire partially affected the forest in Tampa, which regenerated naturally afterward.

In September and October of 2020, bud samples were collected for DNA isolation from a total of 500 adult beech trees from the five populations, from ~100 trees randomly selected at each site (Fig. 1 and S1.1, Supplementary Material).

Bud burst data from Ciocîrlan et al. (2024a) was re-analysed, with a particular focus on the duration and synchrony of spring phenology. Briefly, bud burst stages were recorded for 30 trees per population at a minimum distance of 25 m using the same classification for leaf development as described by Vitasse et al. (2009). The four stages were: 0: at least 50% of the buds in dormancy, 1: at least 50% of the buds swollen and the scales separated, 2: at least 50% of the buds broken and leaves started emerging, 3: at least 50% of the leaves fully developed and petioles extended (further information can be found in Ciocîrlan et al. 2024a). Bud burst stages were recorded 2–3 times per week from March to June 2021 and 2022.

### DNA isolation and genotyping

The DNA was extracted using the DNeasy 96 Plant Kit and DNeasy Plant Mini Kit (Qiagen, Hilden, Germany) following the manufacturer’s instructions. All samples were genotyped at six highly polymorphic genomic microsatellite markers (gSSRs, genomic simple sequence repeats) and six expressed sequence tag microsatellites (EST-SSRs) which are located in expressed regions of the genome and therefore are less polymorphic than those from non-coding regions of the genome (Ellis and Burke 2007). The gSSR markers were sfc0018, sfc0161, sfc1063 and sfc1143 (Asuka et al. 2004), FS3-04 (Pastorelli et al. 2003), mfs11 (Vornam et al. 2004). The EST-SSR markers were FgSI0006 (Burger et al. 2018), FgSI0009, and FgSI0024 (Kubisiak et al. 2009), FS_C1968, FS_C2361 and FS_C7377 (Burger et al. 2018) (Table S2.1 Supplementary Material). PCRs were run in three multiplexes on a Biometra T Professional thermocycler (Analytik Jena, Jena, Germany) following a touch down PCR protocol (S2 and Table S2.2, Supplementary Material). The internal size standard GeneScan™ 500 ROX® (Applied Biosystems, Foster City, CA) was run jointly with the PCR products on a 3130xl Genetic Analyzer (Applied Biosystems, Foster City, CA, USA). Allele sizes were determined using GeneMapper 4.1 (Applied Biosystems, Foster City, CA, USA). Micro-Checker 2.2.3 (Van Oosterhout et al. 2004) was used to check all loci for the presence of null alleles where only one locus (FgSI0006) showed a low null allele frequency.

In addition to microsatellite-based genotyping, a targeted genotyping method based on single primer enrichment technology (SPET) was applied (Scaglione et al. 2019). This method allows for the identification of genome-wide SNP markers via efficient and cost-effective genotyping-by-sequencing of target SNPs. Based on whole-genome sequencing of a subset of 96 samples, ~100k high quality target regions were determined for SPET sequencing by IGATech (IGA Technology Services Srl, Udine, Italy). The SPET sequencing included flanking regions of ±2 kb of the targets to allow for the identification of novel SNPs located within and close to genes. All 500 *F. sylvatica* samples were SPET sequenced and sequencing reads were trimmed and mapped to the chromosome level *F. sylvatica* genome v2 (Mishra et al. 2022). Quality trimming was performed using ERNE v1.4.6 (Del Fabbro et al. 2013) with default parameters. Alignment to the reference genome was accomplished using BWA-MEM v0.7.17 (Li and Durbin 2009) with default parameters. Uniquely aligned reads, defined as reads with a mapping quality ≥10, were selected. Duplicated reads were removed with UMI -tools v1.1.2 (Smith et al. 2017). Variant calling was conducted with Freebayes v1.3.6 (Garrison and Marth 2012). To avoid false calls and variation due to sequencing artefacts the following settings were used: Only sites with at least 6 reads per allele in at least one sample (--min-alternate-count 6), an allele fraction of 0.2 in diploid samples (--min-alternate-fraction 0.2 -p 2) and only two alleles per genotype (--use-best-n-alleles 2) were called. Subsequently, normalization and filtering of variants were performed using bcftools (Danecek et al. 2011). Variants were filtered for a read depth of more than 6x but less than 257x. Loci were considered only if at least half of the samples provided a coverage of 6x or above (N_PASS(FORMAT/GT = = ‘RA’ | | FORMAT/GT = = ‘AA’) > = 1). After this, a total of 838,522 SNPs were retained. In order to identify neutral variants for population genetic analysis, the following filtering steps were applied. First, SNPs located in intergenic regions were determined using SnpEff (version 4.3t, Cingolani et al. 2012). For this, an annotation database based on the *F. sylvatica* reference sequence and gene annotations (Mishra et al. 2022) was built, based on which the effects for each SNP, such as intergenic region variant, missense variant, or intron variant, were inferred. In a second filtering step, SNPs in linkage disequilibrium were filtered out using PLINK (v1.90b6.10) (Purcell et al. 2007) with an ld-window size of 100,000 kb (--ld-window-kb). Retaining unlinked SNPs and one representative SNP per haplotype block yielded a data set of 270,287 SNPs. After filtering out loci with >10% missing data and individuals with missingness >20%, the data set comprised 154,437 polymorphic SNPs. Lastly, in order to identify a unique set of SNPs that are polymorphic in all five populations, the populations program from the Stacks analysis tool set (version 2.2, Rochette et al. 2019) was applied. Based on its output *sumstats*, a list of SNPs with observed heterozygosity >0 in all five populations was prepared, which was then used as a whitelist for vcftools (version 0.1.14, Danecek et al. 2011) to generate a vcf file comprising 51,485 SNPs.

### Population genetic analyses

With the aim of characterizing and visualizing the population genetic structure a Discriminant analysis of principal components (DAPC) implemented in the “adegenet” package (Jombart and Ahmed 2011), was run based on SSR and SNP markers in R (‘R Core Team’ 2023). Genetic diversity parameters and pairwise genetic differentiation at SSR markers were calculated using SPAGeDi 1.5 d (Hardy and Vekemans 2002). We determined the number of alleles (*N*_A_), the expected heterozygosity (*H*_E_) and the allelic richness (*A*_R_) on a standardised sample of 196 gene copies. We tested if the inbreeding coefficient (*F*_IS_) deviated significantly from zero using 10,000 permutations. We also estimated global *F* statistics and pairwise *F*_ST_ values (Weir and Cockerham 1984) between populations and tested for significance using 10,000 permutations. GenAlEx 6.5 (Peakall and Smouse 2012) was used to assess the number of private alleles per population. We used Spearman’s rank correlation coefficient to test for significant correlations between population genetic diversity parameters and elevation in R.

For the SNP markers, we used all 154,437 polymorphic SNPs to characterise the genetic diversity and pairwise population differentiation. VCFtools was used to report the number of polymorphic sites and the number of private alleles per population. Using the package hierfstat in R, we estimated *H*_E_ and the pairwise *F*_ST_ values (Weir and Cockerham 1984) and 95% bootstrap confidence intervals (CI) to assess if pairwise population differentiation based on SNPs was significantly different from zero.

The fine-scale spatial genetic structure (FSGS) was assessed in each of the stands using SPAGeDi. We defined seven distance classes (20 m, 40 m, 80 m, 160 m, 320 m, 640 m, 1400 m, see Appendix 1 SPAGeDi output) and calculated the Loiselle kinship coefficient *F*_ij_ (Loiselle et al. 1995) for all pairs of samples per distance class, using SSR and SNP markers (vcf file comprising 51,485 SNPs polymorphic in all sites). The average pairwise kinship coefficients per distance class were regressed on the logarithm of pairwise spatial distances. To determine the significance of the observed FSGS, the regression slope (*b*) was tested against a random distribution after 1000 permutations of individual locations. The *Sp*-value, indicating the strength of the FSGS, was estimated using the formula *Sp* = -*b*/(1-*F*_1_) (Vekemans and Hardy 2004) where *b* is the regression slope and *F*_1_ refers to the average kinship coefficient in the first distance class (20 m) (Vekemans and Hardy 2004). To characterise the strength of the FSGS we calculated *Sp*-values and the *p*-values of the regression slope. To specifically test for significant differences between locations we estimated mean Jackknife regression slopes *b* and 95% Jackknife confidence intervals (CIs) of *b*. This was done separately for each location and marker type. Non-overlapping CIs were considered to indicate significant differences.

With the aim to compare our results with FSGS estimates from natural populations of *F. sylvatica* growing along an elevational gradient at Mont Ventoux, France, we used the FSGS estimates published by Lander et al. (2021). Based on SSR data from 71 populations Lander et al. (2021) compared the FSGS in old forests (>150 years, named refugial regions) with recently recolonised forests (<150 years). For the comparison, we used the *Sp*-estimates from 23 beech stands from two refugial regions with at least 30 individuals per population along the southern and western slopes of Mont Ventoux (Table S5.1, Supplementary Material). As the maximum sample distance was shorter in stands at Mont Ventoux, we assessed the FSGS and estimated *Sp* in the Romanian populations over a restricted distance range (up to 120 m) for comparison. We tested for significant correlations between *Sp*-values and elevation using the Spearman’s rank correlation coefficient.

### Spring phenology

Based on the bud burst data published by Ciocîrlan et al. (2024a), we calculated the average time (day of the year, DOY) when trees reached bud burst stage 3 (when >50% of leaves were fully developed), as well as the standard deviation (SD) for each population. The DOY were estimated using summarySE from “Rmisc” package (Hope 2013) and plotted using the “ggplot2” package (Wickham et al. 2016) in R. Bud burst duration for each individual was calculated as the number of days from the start of stage 1 (initiation) to the end of stage 3. The overall duration of bud burst for each population was defined as the DOY when the first individual started stage 1 to the DOY when the last individual reached stage 3. We applied Spearman’s rank correlation coefficient to test for significant correlations between the strength of FSGS (*Sp-*values) and the duration of bud burst and the variation (SD) in time of reaching bud burst stage 3, respectively.

## Results

### Genetic diversity and population differentiation

Genetic diversity, as evaluated by *H*_E_, was similar among the five populations. A slightly decreasing trend with increasing elevation for *A*_R_ and the number of private alleles was observed at SSRs and for the number of polymorphic loci and private alleles at SNPs (Table 2, Fig. S4.1, Supplementary Material).

**Table 2:** Overview of genetic parameters of the five *Fagus sylvatica* populations in the Romanian Carpathians using 12 microsatellites and 154,437 Single Nucleotide Polymorphisms.

Population names	Genotyped sample size	Marker	*A*_R_	*N*_A_	Polymorphic sites/ markers	Private alleles	*H*_E_	*F*_IS_	*Sp-*value
Ruia	100	SSR	9.10	9.17	12	3	0.66	0.008	0.00387
	99	SNP			80024	4503	0.16	0.006*	0.00201***
Lupului	100	SSR	9.10	9.08	12	2	0.65	0.010	0.00028
	97	SNP			88804	3993	0.16	0.012*	0.00249***
Solomon	100	SSR	9.60	9.58	12	3	0.66	0.004	0.00832***
	98	SNP			96969	7057	0.16	0.018*	0.00349***
Tampa	100	SSR	9.40	9.42	12	4	0.66	−0.017	0.00387**
	98	SNP			92390	5013	0.16	0.006*	0.00383***
Lempes	100	SSR	9.30	9.33	12	4	0.67	0.023	0.00616***
	100	SNP			97213	7608	0.16	0.006*	0.00443***

*A*_*R*_ Allelic richness, *N*_*A*_ Number of alleles, *H*_*E*_ expected heterozygosity, *F*_*IS*_ individual inbreeding coefficient with significance levels based on permutation tests; Sp-values and significance levels of the regression slope based on permutation tests. Populations are listed from highest to lowest elevation, from top to bottom.

Significance: *P-value < 0.05; **P-value < 0.01; ***P-value < 0.001.

Pairwise population differentiation at SSRs and SNPs was low but mostly significant, ranging from 0–0.020 at SSRs and from 0.007–0.023 at SNPs (Table S3.1 and Table S3.2, Supplementary Material). Both marker types indicated slightly stronger differentiation in particular, between Ruia and Lempes, the highest and lowest elevation populations which are furthest apart from each other (20 km). The DAPC plots revealed a weak population genetic structure at SSRs and a slightly distinct pattern at SNPs, with Ruia and Lempes separated from the other populations. Axis 1 reflected the locations of the populations along the elevation gradient (Fig. S3.1, Supplementary Material).

### Fine-scale spatial genetic structure

**Fig. 2:**
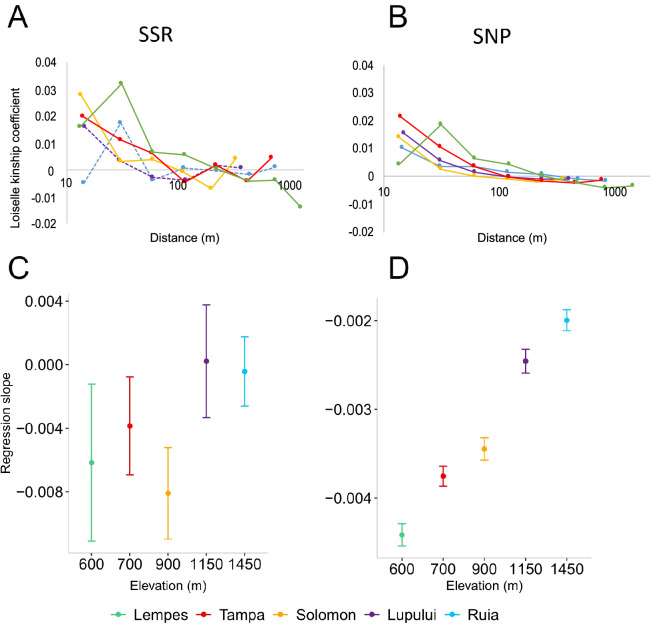
Fine-scale spatial genetic structure (FSGS) in five *Fagus sylvatica* populations along the elevational gradient in the Carpathian Mountains. Pairwise Loiselle kinship coefficient per distance class plotted against the average pairwise distances between trees, using SSR (**A**) and SNP (**B**) markers. Solid lines represent populations with a significant regression slope based on permutation tests. FSGS estimates expressed as mean Jackknife regression slopes *b* and their 95% confidence intervals (CIs), for SSR (**C**) and for SNP markers (**D**). Non-overlapping CIs indicate significant differences between locations.

The strength of FSGS assessed as *Sp*-values ranged from 0.0002 to 0.0083 at SSRs and 0.002 to 0.004 at SNPs and decreased with elevation (Table 2). The p-values of the regression slopes based on permutation tests indicated significant FSGS in the three intermediate and low elevation populations (Solomon, Tampa and Lempes) based on SSRs and in all populations based on SNPs. To assess whether FSGS differed significantly between locations, we specifically compared the mean Jackknife regression slopes (*b*) and their 95% Jackknife CIs for both SSR and SNP markers. Significant differences between populations were detected in particular for SNP markers based on non-overlapping Jackknife CIs (Fig. 2).

With the aim to compare our FSGS results with those published by Lander et al. (2021) for beech populations along an elevational gradient at Mont Ventoux (France) we re-analysed our SSR genotypes. The maximum sample distance between pairs of trees affects the level of FSGS, therefore, we used restricted regression analyses, up to 120 m, similar to the maximum distances in the French populations. As expected, *Sp*-values from restricted regression analyses of our SSR genotypes were higher than our previous estimates (Table S5.1, Supplementary Material). We observed decreasing *Sp*-values with increasing elevation along both elevational gradients in France and Romania. However, the correlation tests between *Sp*-values and elevation were not significant (Fig. S5.1 Supplementary Material).

### Spring phenology

**Fig. 3:**
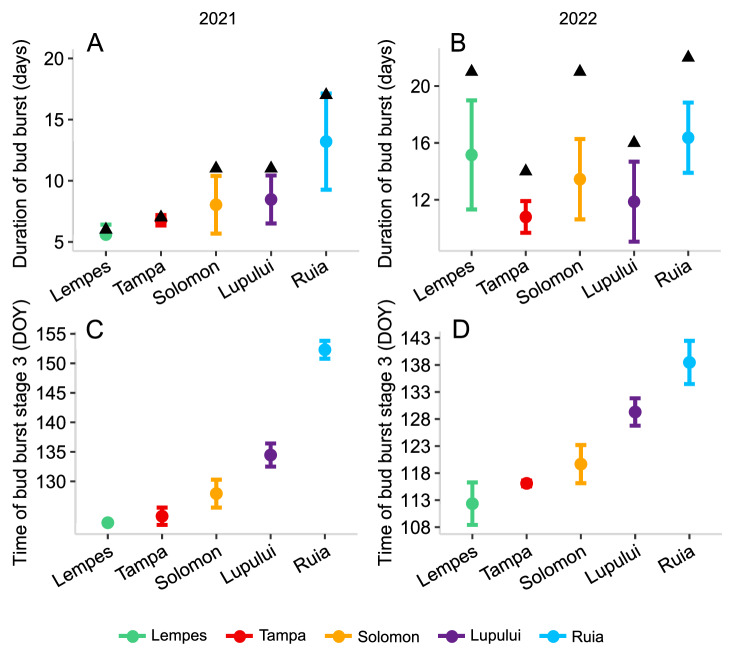
Bud burst in 2021 and 2022 in five *Fagus sylvatica* populations along the elevational gradient in the Romanian Carpathians. Average bud burst duration (defined as number of days between start of stage 1 and start of stage 3) and standard deviation in each *Fagus sylvatica* sample location for 2021 (**A**) and 2022 (**B**). Triangles indicate the overall duration of bud burst from the first individual starting stage 1 until all individuals have reached stage 3. Average time in days when trees reached stage 3 (bud burst stage temporally closest to pollen release) for each sample location in 2021 (**C**) and 2022 (**D**). The locations are plotted from lowest to highest elevation, with increasing elevation indicated from left to right.

Populations located at higher elevations flushed later in both years (Ciocîrlan et al. 2024a) and it took longer until all trees completed the different flushing stages than at lower elevations (Fig. 3). High inter-individual differences in the duration of bud burst indicated less synchrony at high elevations in particular in 2021. In 2022, however, Lempes, the lowest elevation population, exhibited as strong inter-individual differences in spring phenology timing as the highest elevation population. Likewise, within-population synchrony of bud burst stage 3 was strong and similar along the elevational gradient. Correlation tests did not reveal any significant correlations between spring phenology and the strength of FSGS (Fig. S5.2, Supplementary Material).

## Discussion

The steep change in environmental conditions along elevational gradients causes a complex interplay of interspecific competition, demographic processes, gene flow and selection. Here, our aim was to study the neutral genetic variation using SSR and a high number of genome-wide intergenic SNP markers to characterise the genetic diversity and FSGS in five naturally regenerating *F. sylvatica* populations along an elevational gradient in the Romanian Carpathians. We will first discuss the weak population genetic structure and the trend of decreasing genetic variation with increasing elevation. Afterwards, we will discuss the decrease in family structure within populations with increasing elevation and compare our results to previously published data in particular from the Mont Ventoux (Lander et al. 2021).

### Population genetic structure and genetic diversity

The south-eastern Carpathian mountains were recolonised by *F. sylvatica* after the last glacial period ca. 3000–5000 years ago (Magri 2008; Höhn et al. 2021). Recolonisation most likely occurred from refugial locations in the western and southern Carpathians (Willner et al. 2009; Magri et al. 2006) which were admixed with beech trees originating from the main Central European refugium (Gömöry et al. 2020). Our study area is characterised by naturally regenerating beech stands, partly included in natural protected areas. Overall, only a weak population genetic structure was detected. This is in line with previous studies, as *F. sylvatica* is characterised by only three widespread gene pools throughout its distribution range, and populations from the Carpathian Mountains cluster with populations from the Apennines and the Balkan Peninsula (Postolache et al. 2021). At the local to regional scale, wide ranging gene flow (Piotti et al. 2012; Gauzere et al. 2013) typically contributes to low population differentiation and high levels of genetic diversity in beech (Vornam et al. 2004; Buiteveld et al. 2007; Piotti et al. 2012; Rajendra et al. 2014). The most differentiated populations Lempes (lowest elevation) and Ruia (highest elevation) are located at a distance of 20 km indicating isolation by distance along the gradient. All study populations harboured high genetic diversity based on *H*_E_ with comparable levels as observed in other studies (Pluess and Weber 2012; Paffetti et al. 2012; Rajendra et al. 2014; Csilléry et al. 2014; Pluess et al. 2016; Cuervo-Alarcon et al. 2018; Stefanini et al. 2022). However, we found a slight decrease of genetic variation with increasing elevation for the number of polymorphic sites (SNPs), number of alleles (SSRs), and private alleles. These genetic diversity parameters are more difficult to compare directly with other studies as they are strongly affected by the choice of markers and the number of samples. However, they are more sensitive to detect genetic erosion than e.g. *H*_E_ (Hoban et al. 2014). Another study on beech found a similar pattern with lower diversity at high elevations for the percentage of polymorphic sites based on AFLP (Amplified Fragment Length Polymorphism) markers in beech populations from the Montseny Mountains (Spain) spanning an elevational gradient from ca. 992 to 1640 m a.s.l. (Jump and Peñuelas 2007). Comparing our results with those published by Lander et al. (2021) revealed a similar, decreasing trend (although not significant) of genetic diversity (*H*_E_) with increasing elevation along the western slope of Mont Ventoux (Fig. S5.1, Supplementary Material). Also, in other tree species similar patterns of a decreasing trend in genetic diversity with elevation were found, e.g., for *Nothofagus pumilio* (Poepp. & Endl.) Krasser (Premoli 2003) and *Podocarpus parlatorei* Pilg. (Quiroga and Premoli 2007) while other case studies identified different trends with highest genetic diversity at high or intermediate elevations (reviewed in Ohsawa and Ide 2008). Direct comparisons between studies are often hampered by differences, e.g., in latitude, aspect or the elevation range covered by the different gradients. The high elevation populations in this study are located close to the ecological range limit of beech in the Carpathians and therefore characterised as ecologically marginal compared to the populations at lower elevations. This is also reflected by the lower density of beech at high elevations (proportion of beech only ~ 10–20%) indicating that interspecific competition may be strong and the recruitment success may be low. This could result in increased genetic drift compared to lower elevations, and explain the slightly lower levels of genetic diversity.

### Fine-scale spatial genetic structure

The strength of FSGS identified based on SSR markers in the five populations in our study area (*Sp*-values ranging from 0.0002 to 0.0083) was similar to previous studies in natural *F. sylvatica* populations (*Sp*-values ranging from non-significant 0.001 to significant 0.032; Jump and Peñuelas 2007; Chybicki et al. 2009; Jump et al. 2012; Piotti et al. 2013; Lander et al. 2021; Sandurska et al. 2024). These values are typical for wind-pollinated species with zoochorous and gravity dispersed seeds (Vekemans and Hardy 2004; Gelmi-Candusso et al. 2017). However, using both SSR and SNP markers, we observed a decreasing trend in the FSGS with increasing elevation, meaning that high density, low elevation populations showed a stronger family structure than low density, high elevation populations. We found a similar pattern along slopes of the Mont Ventoux based on data published by Lander et al. (2021) (Fig. S5.1, Supplementary Material). FSGS was slightly stronger in populations from Mont Ventoux, however the trends along the elevational gradients were very similar although correlation tests of *Sp*-values with elevation were not significant. Unfortunately, Lander et al. (2021) only used SSR markers and a comparison with our results based on SNPs was not possible with the French populations. However, the high number of genome-wide SNP markers revealed the same trend along the elevational gradient and based on non-overlapping 95% confidence intervals of regression slopes we detected significant differences between all populations in the Romanian Carpathians (Fig. 2D).

Lower recruitment success due to intra- and interspecific competition and harsher environmental conditions may blur the family structure in high elevation locations (Zhou and Chen 2010). Furthermore, pollen dispersal distances are strongly influenced by the canopy density in *F. sylvatica*, where a high density reduces pollen flow distances (Millerón et al. 2012; Gauzere et al. 2013). In our study area, Lempes, the lowest elevation population with high beech density exhibited the strongest FSGS and Ruia, the highest elevation population with lowest beech density and where the forest is characterised by gaps showed the weakest FSGS. This may indicate higher pollen mediated gene flow in high elevations.

In 2021 and 2022, bud burst and hence flowering started later in high elevation compared to low elevations (Ciocîrlan et al. 2024a) as it is typical along elevational gradients. Gauzere et al. (2013) observed higher pollen immigration in high elevation sites from lower elevations at Mont Ventoux which may be due to the temporal difference in female and male flowering (Nielsen and Schaffalitzky de Muckadell 1954), as female flowers in high elevation sites may be receptive when male flowers shed pollen in lower elevations. This could promote higher pollen immigration to high altitude populations and contribute to a weaker FSGS. Furthermore, our results of bud burst duration indicated a trend from more to less synchrony within-populations from low to high elevations only in 2021. However, the within-population synchrony of pollen release, as indicated by the temporal variability of reaching bud burst stage 3, appeared to be similar in all locations. We could not detect any significant correlations between spring phenology traits and the level of FSGS. However, future studies could explore the effects of flowering synchrony and autocorrelations of flowering traits within populations over several years on the level of FSGS using larger numbers of phenotyped trees, with a representative distribution at different distance classes.

## Conclusions

A better understanding of the natural variation in FSGS and genetic diversity along environmental gradients is crucial to inform forest and conservation management in particular in the context of climate change. Our results shed new light on the variation of FSGS and genetic diversity in naturally regenerating beech populations. The direct comparison between SSRs and genome-wide intergenic SNPs highlighted the advantage of a high number of markers to reveal accurate estimates of FSGS and in detecting significant differences among populations which would otherwise remain inconspicuous.

Ecologically marginal high elevation populations showed slightly lower genetic diversity and weaker FSGS than low elevation populations. A reduced density, possibly due to harsher conditions and lower recruitment success can enhance genetic drift at high elevations. Furthermore, the more open forest structure might promote longer ranging pollen dispersal via wind compared to denser low elevation stands blurring the FSGS. However, we could not demonstrate a significant correlation between synchrony of within-population spring phenology and the level of FSGS, likely because FSGS patterns result from a combination of factors. Interannual variation in environmental conditions such as wind strength and direction, humidity, temperature, and frost events may influence reproductive timing, dispersal dynamics, seedling establishment and mortality, thereby jointly shaping the FSGS. Further studies along elevational gradients in beech populations from other regions and in species with similar life history traits are needed to test if these are general trends.

## Supplementary information

Supplementary Material

Appendix 1

## Data Availability

GPS coordinates, microsatellite and SNP genotype data are available from the Göttingen Research Online repository: 10.25625/680ZPM.
