# Supplementary material for: Genetic diversity and fine-scale spatial genetic structure of European beech populations along an elevational gradient
Source: Heredity (Edinb). 2025 Jun 26;134(8):451–60. doi: 10.1038/s41437-025-00776-8 (PMC12316929; doi:10.1038/s41437-025-00776-8)
Supplement: Supplementary file 1 — Supplementary Material [file 41437_2025_776_MOESM1_ESM.docx]

# **Supplementary material**

**Genetic diversity and fine-scale spatial genetic structure of European beech populations along an elevational gradient**

Ourania Grigoriadou-Zormpa^1,2^, Selina Wilhelmi^1,2^, Boban Vucetic^1^, Mihnea-Ioan-Cezar Ciocîrlan^3,4^, Markus Mueller^1,2^, Elena Ciocîrlan^3^, Alexandru Lucian Curtu^3^, Mehdi Ben Targem^5^, Henning Wildhagen^5^, Oliver Gailing^1,2,6*^, Katharina B. Budde^1,7^*

1. Faculty of Forest Sciences and Forest Ecology, Forest Genetics and Forest Tree Breeding, University of Göttingen, Büsgenweg 2, 37077 Göttingen, Germany
2. Center for Integrated Breeding Research (CiBreed), University of Göttingen, Von-Siebold-Str. 4, 37075 Göttingen, Germany
3. Faculty of Silviculture and Forest Engineering, Transilvania University of Brașov, Sirul Beethoven 1, 500123 Brașov, Romania
4. National Institute for Research and Development in Forestry (INCDS) Marin Drăcea, Brașov Station, Romania
5. HAWK University of Applied Sciences and Arts, Faculty of Resource Management, Büsgenweg 1a, 37077, Göttingen, Germany
6. Center of Biodiversity and sustainable Land Use (CBL), University of Göttingen, Büsgenweg 1, 37077 Göttingen, Germany
7. Northwest German Forest Research Institute, Prof.-Oelkers-Straße 6, 34346, Hann Münden, Germany

*: corresponding authors: katharina.budde@nw-fva.de; ogailin@gwdg.de

**S1 Sample distribution**


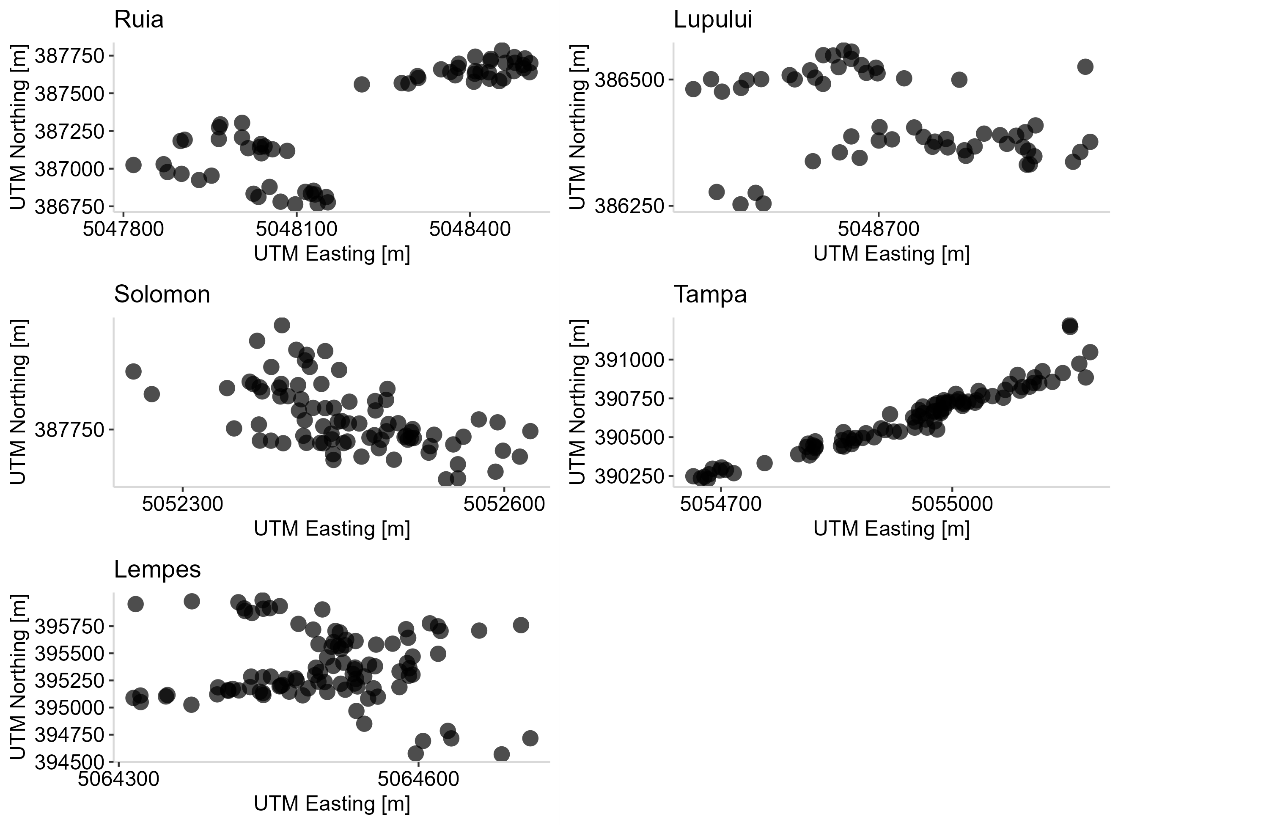


Figure S1.1 Spatial distribution of sampled Fagus sylvatica trees in each location along the elevational gradient in the Romanian Carpathians.

**S2 SSR genotyping**

DNA concentration was measured using a Qubit 3.0 fluorometer (Invitrogen, NY, USA) and diluted to ca. 10 ng/µl. Each PCR mix was prepared consisting of 1 μl diluted DNA (ca. 10 ng/µl), 10 x 1.5 μl PCR buffer B (Solis BioDyne, Tartu, Estonia), 1.5 μl MgCl_2_ (25 mM), 1 μl dNTPs (2.5 mM each), 1 μl forward primer (5 pmol/ μl), 1 μl reverse primer (5 pmol/ μl), and 0.2 μl Taq polymerase (5 units per μl, Hot Fire Pol® DNA Polymerase, Solis BioDyne, Tartu, Estonia). Water was added to reach the final reaction volume of 14 μl and the PCR was run in a Biometra T Professional thermal cycler (Analytik Jena, Jena, Germany) following a standard protocol (Table S2, Supplementary Material). For the microsatellite fragment size determination, 1270 μl of HiDi formamide (Applied Biosystems, CA, USA) were mixed with 1.6 μl of the internal size standard GeneScan™ 500 ROX® (Applied Biosystems, Foster City, CA). For each well, 12 μl of the mix was added to 2 μl of diluted PCR product. Prior to genotyping on a 3130xl Genetic Analyzer (Applied Biosystems, Foster City, CA, USA), the mixture was denatured for three minutes at 95 °C. Allele sizes were determined using GeneMapper 4.1 (Applied Biosystems, Foster City, CA, USA). Cumulative distributions of allele sizes were plotted for each marker using Excel 11 (*Microsoft Office*, 2021), clusters of allele sizes were identified and sorted into representative sizes through manual binning. Micro-Checker 2.2.3 (Van Oosterhout *et al.*, 2004) was used to check all loci for the presence of null alleles where only one locus (FgSI0006) showed a low frequency of null alleles.

Table S2.1 Details of the nuclear microsatellite markers (*EST-SSRs), fluorescence labels and multiplexes.

| Locus | Motive | Size (bp) | Forward primer (5`-3`) | Reverse primer (5`-3`) | Fluorescence label | Multiplex |
| --- | --- | --- | --- | --- | --- | --- |
| sfc0018 | (AG) 17 | 151-188 | GAAGCAGAGCATTGTATTGG | CATCTGTTTCAGTTCTGTAAAGG | HEX | I |
|  |  |  |  |  |  |  |
| sfc0161 | (AG) 22 | 104-148 | AAGCTCCACGATTCATTC | GCTGGAGTTGCTCTAAGTC | 6-FAM | I |
|  |  |  |  |  |  |  |
| sfc1063 | (CT) 13 | 187-211 | TTTCCAACTACAACTTCATTG | AGTGCTCGCATCGTATG | 6-FAM | I |
|  |  |  |  |  |  |  |
| sfc1143 | (AG) 21 | 102-136 | TGGCATCCTACTGTAATTTGAC | ATTCCACCCACCATCTGTC | HEX | I |
|  | (GCT) 5 |  |  |  |  |  |
| FS3-04 | (GTT) 3 | 191-206 | AGATGCACCACTTCAAATTC | TCTCCTCAGCAACATACCTC | HEX | II |
|  | (GCT) 6 |  |  |  |  |  |
| mfs11 | (AG) 10 | 130-150 | GGGGGTGGTTTCAAGTTTC | GAAGCCAATTATCACACCAAAAG | HEX | II |
|  |  |  |  |  |  |  |
| FgSI0006 * | (TGT) n | 259-264 | TTAACACCGCGGTAGAGACC | GCTCCAAGCTCTTGCTCACT | 6-FAM | III |
|  |  |  |  |  |  |  |
| FgSI0009 * | (ACC) n | 214-217 | TACCCATGCCCATATCCAGT | GGAAAGAAGAAGGGTGGAGG | HEX | III |
|  |  |  |  |  |  |  |
| FgSI0024 * | (TCG) n | 166-301 | GAATCGTCGGAATCGTTGTC | CGGTCGAGGATGATGACTTT | 6-FAM | III |
|  |  |  |  |  |  |  |
| FS_C1968 * | (TC) n | 299-301 | ATCGTTTCCACTTCCTCCGG | TTTCATGCACCCTCTCTAGG | 6-FAM | III |
|  |  |  |  |  |  |  |
| FS_C2361 * | (GAA) n | 196-205 | AGGTCCTTCAGTTTGGGAGC | ATTCCCATGCATCAAAATCC | 6-FAM | III |
|  |  |  |  |  |  |  |
| FS_C7377 * | (GAT) n | 139-166 | AATCGGACGGTCCATAGTGC | AGATCCGAGCTCAACTCACC | HEX | III |

Table S2.2 Details of the Touch down PCR protocol used for amplification of nuclear microsatellites for the Fagus sylvatica samples. Min, minutes.

|  | Run 1 | Time | Cycles |
| --- | --- | --- | --- |
| Taq polymerase activation | 95°C | 1 min |  |
|  |  |  |  |
| Denaturation | 94°C | 1 min |  |
| Annealing | 60°C (-1°C per cycle) | 1 min | 10 |
| Elongation | 72°C | 1 min |  |
|  |  |  |  |
| Denaturation | 94°C | 1 min |  |
| Annealing | 55°C | 1 min | 25 |
| Elongation | 72°C | 1 min |  |
|  |  |  |  |
| Extension | 72°C | 20 min |  |
| Hold | 16°C | ∞ |  |

**S3 Population differentiation and population genetic structure**

Table S3.1 Pairwise F_ST_ and p-values (above diagonal) for the Romanian Fagus sylvatica populations for SSRs. Significant values are indicated in bold

| F_ST_/P-Value | Ruia | Lupului | Solomon | Tampa | Lempes |
| --- | --- | --- | --- | --- | --- |
| Ruia |  | **0.0019** | **<0.0001** | **<0.0001** | **<0.0001** |
| Lupului | **0.0044** |  | **0.0229** | 0.0918 | **<0.0001** |
| Solomon | **0.0079** | **0.0026** |  | 0.4547 | **<0.0001** |
| Tampa | **0.0066** | 0.0016 | 0 |  | **<0.0001** |
| Lempes | **0.0203** | **0.0169** | **0.0110** | **0.0139** |  |

Table S3.2 Pairwise *F*_ST_ for the Romanian *Fagus sylvatica* populations for SNPs. The upper diagonal presents bootstrapped 95% confidence intervals (lower - upper limit) for each pairwise *F*_ST_ comparison. All values are significant as the confidence intervals do not overlap with zero.

| F_ST_/P-Value | Ruia | Lupului | Solomon | Tampa | Lempes |
| --- | --- | --- | --- | --- | --- |
| Ruia |  | 0.0227-0.0233 | 0.0107-0.0112 | 0.0155-0.0161 | 0.0192-0.0197 |
| Lupului | 0.023 |  | 0.0181-0.0185 | 0.0085-0.0089 | 0.0182-0.0186 |
| Solomon | 0.011 | 0.018 |  | 0.0071-0.0074 | 0.0094-0.0097 |
| Tampa | 0.016 | 0.009 | 0.007 |  | 0.0132-0.0136 |
| Lempes | 0.020 | 0.018 | 0.010 | 0.013 |  |


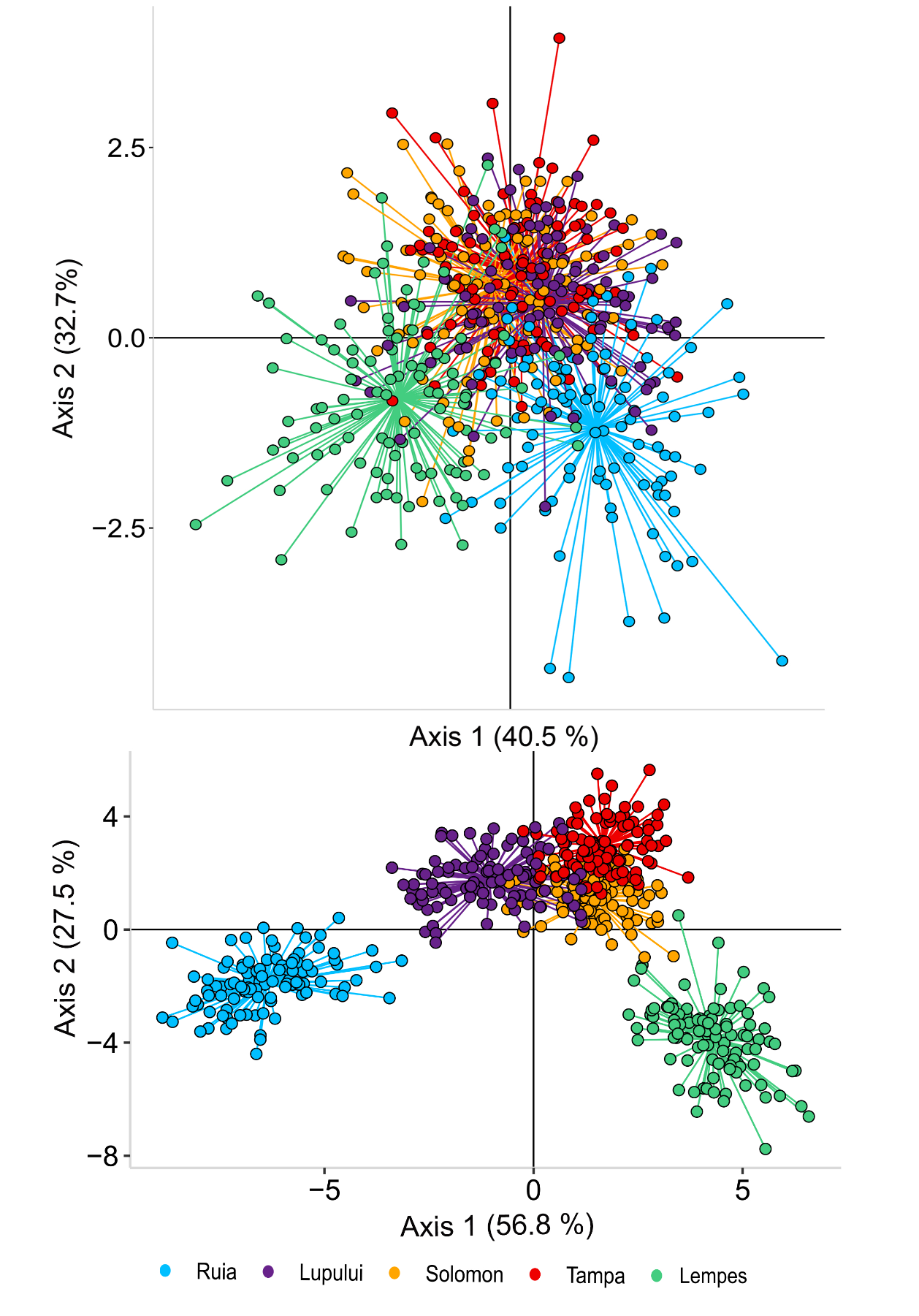


Figure S3.1 Discriminant analysis of principal components (DAPC) plots of the five Romanian Fagus sylvatica populations using SSR (above) and SNP (below) markers.

**S4 Genetic diversity**


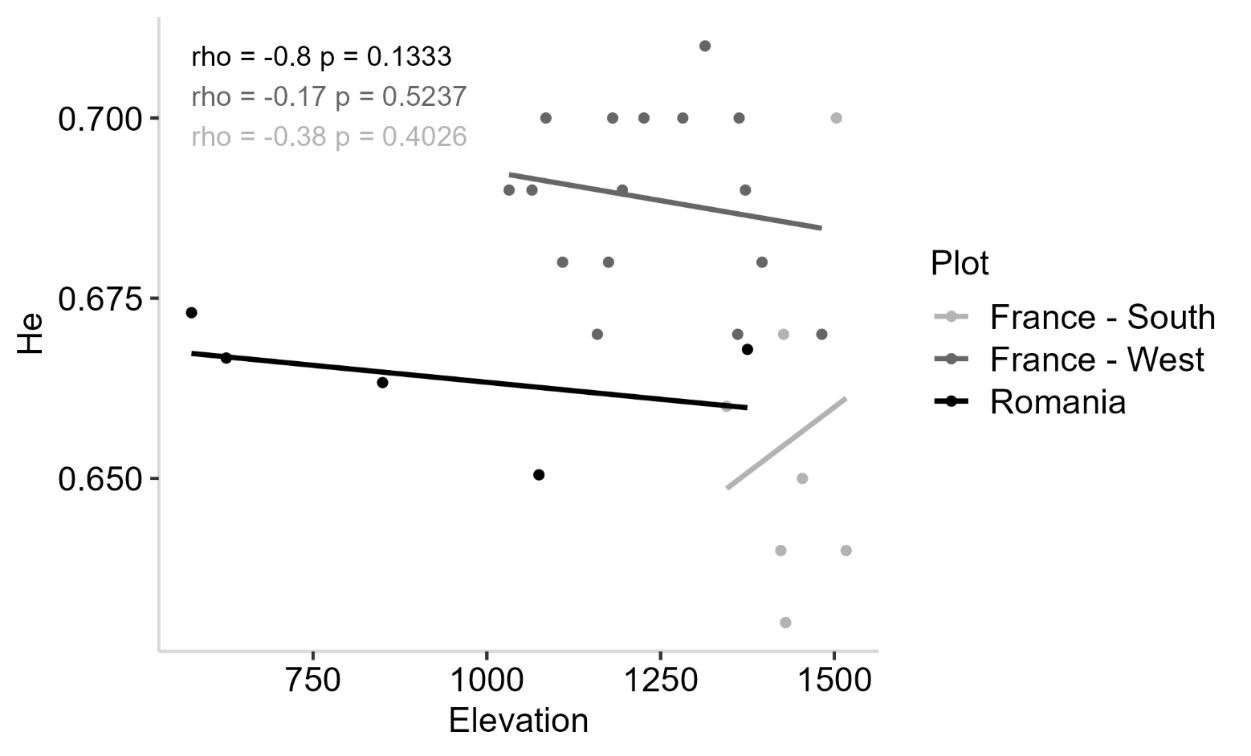


Figure S4.1 Correlation of genetic diversity (He) and elevation for Fagus sylvatica populations from the Romanian Carpathians and refugial locations at Mont Ventoux, France using SSR markers.

**S5 Fine scale spatial genetic structure at SSRs**

*Table S 5.1:* Fine-scale spatial genetic structure assessed as *Sp*-values for the 5 Romanian (this study) and 23 French populations from Western (W) and Southern (S) slopes at Mont Ventoux, France (Lander et al., 2021). *Sp*-values for Romania were estimated using a restricted distance range to align with the distance range at Mont Ventoux. Significance is indicated by * for p < 0.05, ** for p< 0.01, *** for p< 0.001 and ns, non-significant.

| *Location spatial genetic structure assessed as* Sp*-values on SSR markers for the 5 Romanian (this study) and 23 French populations from Western (W) and Southern (S) slopes at Mont Ventoux, France (Lander* et al*., 2021).* Sp *values for Romania were estimated using a restricted distance range to align with the distance range at Mont Ventoux. Significance is indicated by * for p < 0.05, ** for p< 0.01, *** for p< 0.001 and ns, non-significant.*Location | Elevation [m] | Country | Number of samples | *Sp*-value | Significance level |
| --- | --- | --- | --- | --- | --- |
| Ruia | 1400 | Romania | 100 | 0.008 | ns |
| Lupului | 1100 | Romania | 100 | 0.005 | ns |
| Solomon | 900 | Romania | 100 | 0.010 | ** |
| Tampa | 700 | Romania | 100 | 0.017 | *** |
| Lempes | 550 | Romania | 100 | 0.015 | ** |
| S_1101 | 1427 | France | 30 | 0.028 | * |
| S_1225 | 1345 | France | 30 | 0.027 | * |
| S_1250 | 1423 | France | 30 | 0.065 | * |
| S_5 | 1517 | France | 30 | 0.018 | * |
| S_901 | 1454 | France | 30 | 0.014 | ns |
| S_905 | 1430 | France | 30 | 0.027 | * |
| S_985 | 1503 | France | 30 | 0.041 | * |
| W_221 | 1282 | France | 40 | 0.005 | ns |
| W_229 | 1314 | France | 41 | 0.015 | * |
| W_257-1 | 1065 | France | 31 | 0.028 | * |
| W_257-2 | 1032 | France | 184 | 0.026 | * |
| W_260 | 1085 | France | 30 | 0.004 | ns |
| W_263 | 1181 | France | 42 | 0.011 | * |
| W_313 | 1109 | France | 30 | 0.023 | * |
| W_362-1 | 1159 | France | 40 | 0.030 | * |
| W_362-2 | 1175 | France | 42 | 0.029 | * |
| W_384 | 1363 | France | 296 | 0.009 | * |
| W_435 | 1482 | France | 40 | 0.001 | ns |
| W_484 | 1372 | France | 36 | 0.012 | * |
| W_B | 1361 | France | 30 | 0.041 | * |
| W_C | 1396 | France | 30 | 0.042 | * |
| W_N2 | 1195 | France | 99 | 0.015 | * |
| W_N3 | 1226 | France | 31 | 0.007 | ns |


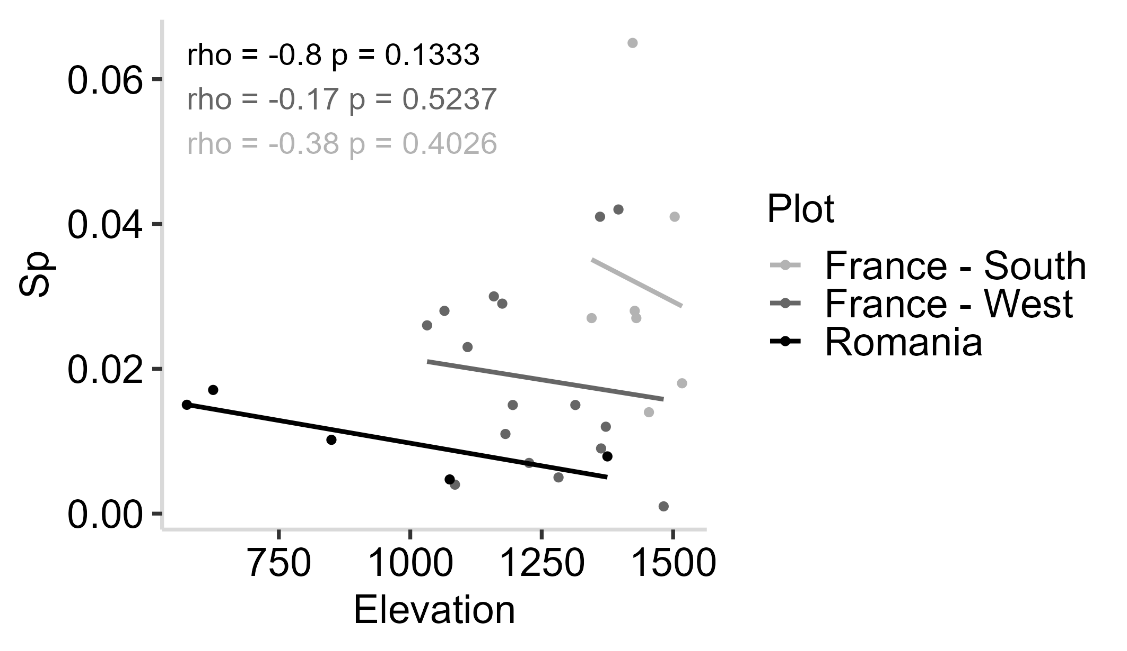


Figure S5.1 Correlation tests of the fine-scale spatial genetic structure assessed as Sp-values for the 5 Romanian (this study) and 23 French populations from Western (W) and Southern (S) slopes at Mont Ventoux, France (Lander et al., 2021). Sp-values for Romania were estimated using a restricted distance range to align with the distance range at Mont Ventoux. Significance is indicated by * for p < 0.05, ** for p< 0.01, *** for p< 0.001 and ns, non-significant.


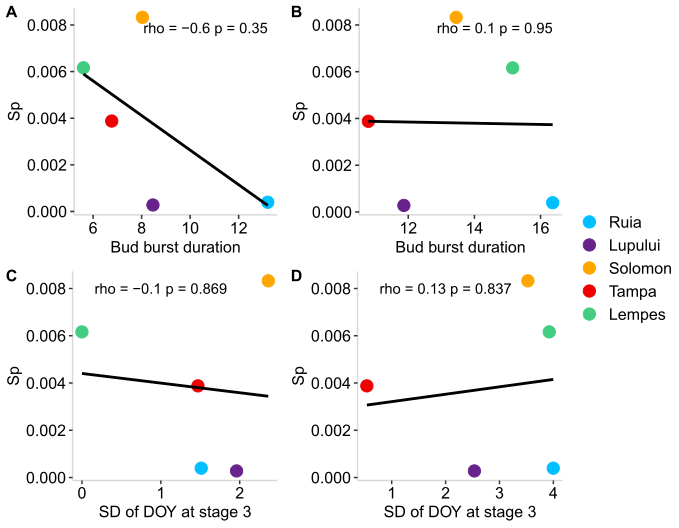


Figure S5.2 Correlation tests of strength of FSGS (Sp-values) and the average bud burst duration in days in 2021 (A) and 2022 (B), and the temporal variability (SD, standard deviation; DOY, day of the year) in reaching bud burst stage 3 for each population in 2021 (C) and 2022 (D).
